# Wood smoke particles elicit events associated with adverse effects in human lung epithelial cells

**DOI:** 10.3389/ftox.2026.1671925

**Published:** 2026-03-20

**Authors:** Deedee Romo, Finnegan Friday, Michael L. Armstrong, Nichole Reisdorph, Vincent Conrad, Marie J. Andales, Jenny Chen, Kaila Cho, Brad L. Upham, Stephen Brindley, Robert C. Canfield, Jared M. Brown, John Volckens, Alison K. Bauer

**Affiliations:** 1 Department of Environmental and Occupational Health, Colorado School of Public Health, University of Colorado Anschutz Medical Campus, Aurora, CO, United States; 2 Department of Pharmaceutical Sciences, Skaggs School of Pharmacy and Pharmaceutical Sciences, University of Colorado Anschutz Medical Campus, Aurora, CO, United States; 3 Department of Mechanical Engineering, Colorado State University, Fort Collins, CO, United States; 4 Department of Pediatrics and Human Development, Michigan State University, East Lansing, MI, United States

**Keywords:** BEAS-2B, connexin, gap junction intercellular communication, human bronchial epithelial cell line, inflammatory mediators, lung, wildfire, wood smoke particles

## Abstract

Exposure to wildfire smoke particulate matter (PM) is increasing around the world due to unprecedented wildfires. Numerous adverse health effects are associated with wildfire smoke PM exposures, including an increased risk of developing lung cancer in wildland firefighters. However more research is needed to fully comprehend the mechanisms involved in response to these exposures. We specifically focused on determining the effects of Douglas fir wood smoke particles (WSP) on several critical cellular events, also known to be included as hallmarks of cancer, on a bronchial epithelial cell line (BEAS-2B). The endpoints studied were pro-inflammatory cytokines/bioactive lipids and dysregulation of gap junctional intercellular communication at noncytotoxic concentrations of WSP. Polycyclic aromatic hydrocarbons (PAHs) were identified in WSP using gas chromatography-mass spectrometry (GC/MS), and WSP increased the mRNA levels of the PAH metabolizing enzymes *CYP1A1* and *CYP1B1*. Levels of mRNA expression of the pro- inflammatory markers *TNF*, *IL-6*, *COX-2*, and *IL-8*, were significantly elevated above the control vehicle at 5 μg/mL WSP. IL-6 secretion was also significantly increased above the control vehicle at 5 μg/mL WSP. Additionally, there was a significant decrease in the expression of gap junction genes (*GJA1* and *GJB2*) along with decreased activity of gap junctional intercellular communication in response to 5 μg/mL WSP. Parthenolide, a strong pan-anti-inflammatory and anti-cancer compound, prevented WSP-induced dysregulation of gap junction activity and *TNF* mRNA expression. Lastly, epiregulin, a known growth factor upregulated in premalignant stages of lung cancer specifically during tumor-promoting inflammation, was also significantly elevated above control in response to 5 μg/mL WSP. These early results support a link between inflammation and gap junctions and provide a critical new mechanistic understanding of how WSP contribute to early adverse events in a lung cell line along with the potential to prevent these adverse outcomes with interventions such as parthenolide.

## 1 Introduction

In the U.S., wildland fires are now the largest source of particulate matter (PM) emissions to the atmosphere (Environmental Proection Agency, 2022). Recent and unprecedented wildfires in the U.S., Canada, Australia, South America, and Europe provide evidence that wildfires are increasing in incidence and are now a global concern for human health (Xu et al., 2020). Human exposure to wildfire PM has been associated with numerous cardiovascular and respiratory health effects including impaired lung function, asthma, chronic obstructive pulmonary disease, and cancer (Navarro et al., 2019b; Xu et al., 2020; Weichenthal, 2025). Epidemiologic research suggests that wildland firefighters (i.e., those exposed to primary emissions) have a 43% increased rate of lung cancer over a 25 y span (Navarro et al., 2019b). A significant, positive association also exists between exposure to ambient PM_2.5_ and lung adenocarcinoma (LUAD) development (the most prevalent form of lung cancer) in those individuals with EGFR mutations (Hill et al., 2023; Diaz-Gay et al., 2025). Only a few epidemiologic or toxicology studies exist in support of wildfire smoke as a carcinogen (Korsiak et al., 2022; Navarro et al., 2019b; Ye et al., 2022). Thus, understanding the potential mechanisms driving these wildfire exposure-induced responses is urgently needed to investigate the long-term effects of these exposures on lung health, such as lung cancer.

Air pollution from wildland fires is a complex mixture of fine and ultrafine particulate matter, carbon monoxide, nitrogen oxides, and volatile organic compounds (Xu et al., 2020). Wildfire smoke PM, which is considered the primary driver of smoke toxicity, is a mixture of organic compounds, elemental carbon, water-soluble ions, and transition metals (Trieu et al., 2024). Among this complex organic mixture, polycyclic aromatic hydrocarbons (PAHs) are the most studied class, owing to their prevalence in combustion-derived PM, their toxicity, and their carcinogenic potential (IARC, 2010; Holme et al., 2023). Studies have reported on community and wildland firefighter exposure to PAHs and their biologic metabolites (Adetona et al., 2017; Navarro et al., 2019a; Grunfeld et al., 2024). Our previous studies examining the premalignant effects of PAHs in *in vivo* and *in vitro* lung models (Bauer et al., 2024; Bauer et al., 2022; Romo et al., 2019; Siegrist et al., 2019) led us to investigate if premalignant events in lung cells occur in response to a wildfire PM surrogate (wood smoke particles, WSP) since little is known about these early events in the lung. Previous literature supports the use of WSP exposure as a surrogate to wildfire smoke PM (Corsini et al., 2013; Noah et al., 2023). Studies observed an increase in adverse cellular effects in response to primary (fresh) WSP, including increased oxidative stress (Ghio et al., 2012; Dilger et al., 2016) and cytokine secretion (Bolling et al., 2012; Totlandsdal et al., 2014; Corsini et al., 2013) in lung and immune cells.

The endpoints we evaluated are considered hallmarks of cancer (Hanahan and Weinberg, 2011) and have been investigated in our laboratories and others (Bauer et al., 2018; Nahta et al., 2015; Romo et al., 2019; Siegrist et al., 2019; Unver et al., 2018). Tumor promoters do not themselves elicit tumors unless preceded by an initiating event such as another carcinogen or virus that causes an oncogenic mutation (Trosko et al., 2004; Trosko and Upham, 2005; Upham and Trosko, 2006; Tennant, 1999). Thus, our studies are not evaluating frank tumor development but rather evaluating those events that can occur due to the WSP alone (i.e, as a promoter). Tumor promoting inflammation is a critical component in the early stages before cancer development (Hanahan and Weinberg, 2011). We previously observed pro-tumorigenic inflammatory cytokines (e.g., IL-6) and pro-inflammatory lipid mediator pathways (e.g., COX-2, PGE_2_) in response to individual and mixtures of PAHs (Bauer et al., 2018; Osgood et al., 2017; Siegrist et al., 2019). Numerous other groups have reported pro-tumorigenic inflammatory cytokine pathways, such as IL-6, as potential intervention targets (Unver et al., 2018). However, several of these same inflammagens (IL6, TNF) have been observed in other lung diseases, such as COPD (Dawson et al., 2021; Caramori et al., 2014), thus these inflammatory mediators are not solely pro-tumorigenic and could be indicators of other lung disease potential. Additionally, reduced gap junctional intercellular communication (GJIC) is a component of the evasion of growth suppression, a key hallmark of the tumor promotion stage (Nahta et al., 2015; Hanahan and Weinberg, 2011). Gap junctions, composed of connexins (CX), are intercellular channels that allow for cell-cell communication, of which suppression can alter the homeostatic balance favoring uncontrolled cell proliferation, decreased differentiation and apoptosis that is common to diseases such as cancer (Johnson and Koval, 2009; Avanzo et al., 2004). CXs 43 (GJA1) and 26 (GJB2) are components of gap junction channels expressed in multiple lung cell types, including epithelial cells such as the BEAS-2B cells used in our study (Johnson and Koval, 2009; Avanzo et al., 2007).

In these studies, we provide evidence in a human lung cell line that WSP induce changes in mRNA expression of specific cytokines, a bioactive lipid pathway, connexins, and enzymes involved downstream of PAH exposures, IL-6 secretion, GJIC activity, and a known growth factor linked to LUAD (epiregulin). These results will lead to a more mechanistic understanding of WSP (wildfire surrogate)-induced adverse lung cell events. Our overall hypothesis is that wood smoke particles elicit pro-inflammatory and cell-cell communication events associated with premalignancy, a stage prior to any tumor development, in human lung epithelial cells.

## 2 Materials and methods

### 2.1 Generation of wood smoke particles from Douglas fir

Primary (fresh) wood smoke particles (WSP) were generated under a custom-designed emission hood from the combustion of Douglas fir wood, the most common tree species found in Western U.S. forests (Stanke et al., 2021; Eden et al., 2023). The Douglas fir wood was purchased from Sears Trostel Lumber and Millwork (Fort Collins, CO), harvested from Northern Colorado, and milled similarly to our previous publication (van Zyl et al., 2019). The milled wood used for these studies was at approximately 15% moisture content. A schematic and photo of the experimental setup at Colorado State University for the wood-burning set-up is found in Supplementary Figures 1 and 2. The modified combustion efficiency averaged 96.8% (± 0.8%) for the particle generation, but ranged from 0.9% to 0.99%, reflecting a mix of flaming and smoldering combustion that occurred during a given burn experiment. The temperature of the flame was not measured, as the flames were transient and highly variable over time. Further, thermocouple-based temperature measurements of open wood combustion are subject to bias from radiant energy transfer via the coal bed. However, mean exhaust temperatures were measured in the plume above the flame, which ranged from 350 to 500 °C. The wood burned through both flaming and smoldering phases. PM_2.5_ samples were collected on 47 mm PTFE membrane filters (46.2 mm with support ring, 2 µm pore size, Tisch Scientific, Cleveland, OH, USA) with URG cyclone (16.7 LPM, 2.5 µm, NC, USA) attached to an Alicat mass flow controller (Tucson, AZ, USA) and a vacuum pump. Teflon filters were weighed before and after WSP collection. The filter membrane was separated from the support ring and then placed in a pre-weighed glass vial. To the vial, extraction solvents were sequentially added as follows: 1 mL of methanol, 1 mL of acetone, and 1 mL hexane. The vial was capped, agitated in a vortex mixer for 1 min and then sonicated for 5 min. The filter membrane was removed from the vial and the remaining extract dried under a stream of nitrogen. The vial containing the dried extract was re-weighed to determine the amount of accumulated PM_2.5._ A control vial was also provided from an air filtered control sample from 47 mm PTFE membrane filter for analysis. Prior to use, endotoxin-free dH_2_O (ThermoFisher) was added to the vial to generate a 10 mg/mL concentration followed by sonication using a sonic dismembrator (Fisher Scientific, Model 705) at 90 amps for 5 min. The stock was then aliquoted and stored at −80 °C until use. Prior to each study, an aliquot of the WSP was placed under a UV hood for 24 h to inactivate any potential endotoxin contamination followed by sonication for another 5 min in the sonic dismembrator at 90 amps. A dilution was then made into the DMEM medium directly (5–100 µg/mL) and vortexed prior to treating the human bronchial epithelial cell line (BEAS-2B).

To measure particle size, we used Dynamic Light Scattering (DLS) with a 1:500 dilution of the 10 mg/mL stock following 5 min in the sonic dismembrator at 90 amps and vortexing the sample. The particle size for the wood smoke particles used had a Z-average of 1969 nm ± 774 (SD) with the primary peak size at 185.1 nm ± 92.25 (SD). These results indicate that there are some large particles or aggregates but the majority of the particles average 185.1 nm in size. In addition, the Pierce Chromogenic Endotoxin Quant Kit was used with the low standards from 0.01–0.1 EU/mL endotoxin to verify that there was no detectable endotoxin in the same WSP concentrations used for these studies (5 and 25 µg/mL WSP). We did not observe any detectable endotoxin in our samples.

### 2.2 BEAS2B culturing and experimental design

An immortalized, normal human bronchial epithelial cell line (BEAS-2B, ATCC, Manassas, VA) of < 12 passages were maintained at 37 °C and 5% CO_2_ with standard aseptic procedures. The cells were cultured in Dulbecco’s Modified Eagle Medium (DMEM, Invitrogen, Waltham, MA) media supplemented with 10% (v/v) fetal bovine serum (FBS) (Sigma-Aldrich) and 1% (v/v) antibiotic-antimycotic (Invitrogen). The BEAS-2B cells are a common cell line used for environmental lung toxicant studies, such as PAHs, nanomaterials, and cigarette smoke (Bauer et al., 2022; Feng et al., 2012; Shetty et al., 2012; Siegrist et al., 2014) and are an accepted model for toxicant studies evaluating premalignancy. BEAS-2B cells are not transformed and according to the ATCC and Sunaga et al., these cells have wildtype EGFR, KRAS, BRAF, and P53, common LUAD driver mutations (Sunaga et al., 2013). For the cytotoxicity assays, RNA expression, and the IL-6 ELISA (see Supplementary Material), BEAS-2B cells were treated in 2.5% FBS DMEM medium and treated for 24 h with the WSP or vehicle control (endotoxin-free dH_2_O). For parthenolide treatment, cells were pretreated with parthenolide (Tocris; 5 μM) for 1 hr prior to WSP exposure in 2.5% FBS in DMEM medium. Parthenolide, a plant-derived sesquiterpene lactone, is pan-anti-inflammatory and blocks several transcription factor pathways (Ghantous et al., 2013); it also has other anticancer properties (Sztiller-Sikorska and Czyz, 2020). For the GJIC assays, BEAS-2B cells were first serum deprived (0%) in DMEM medium for 24 hr prior to another 30 min-24h treatment with the WSP, control, and/or parthenolide treatment in serum-free DMEM media, similar to the method described by (Bauer et al., 2022).

The dose range of the WSP used for these studies was based on several published papers using WSP (ranging from 12.5- 200 μg/mL) (Bolling et al., 2012; Brocke et al., 2022; Corsini et al., 2013; Gupta et al., 2021) as well as ranges for wildfire PM exposures in wildland firefighters. Several published papers determined that the exposure levels for these firefighters can be as high as 78.2–115.2 ng/cm^2^ (Swiston et al., 2008; Naeher et al., 2007). Thus, our primary dose of 5 μg/mL (or 1.136 μg/cm^2^) is ∼10 times higher than exposures to firefighters at a wildland fire. However, these studies did not take into account cumulative lifetime doses for these firefighters, thus making our chosen dose environmentally relevant.

### 2.3 Cytotoxicity assays

The manufacturer’s instructions were followed for both assays, the CellTiter 96 Aqueous One Solution Cell Viability assay (MTS assay, Promega, Madison, WI) and the CyQUANT™ LDH Cytotoxicity Assay Kit (C20300; Invitrogen, Waltham, MA), BEAS-2B cells were grown to confluence in 96 well cell culture plates, followed by WSP treatment. The assays were run after 24 h treatment with the WSP.

### 2.4 Quantitative reverse transcriptase PCR (qRT-PCR)

BEAS-2B cells were treated with 5 μg/mL WSP for 24 hr for all treatment groups including with inhibitors. RNA was isolated using the Macherey-Nagel Nucleospin RNA II kit (Clontech Laboratories, Mountain View, CA) following their kit specifications. One microgram of total RNA was reverse transcribed to cDNA (Bauer et al., 2024). mRNA analysis was done using standard RT-PCR with Sybr green master mix (Applied Biosystems, Foster City, CA, USA) and amplified with primers specific to the genes of interest using a QuantStudio 3 Real time PCR (Applied Biosystems, Waltham, MA). The comparative CT method was used where samples were normalized to the expression of 18S rRNA (Bauer et al., 2017). Sequences for the primers are found in Supplementary Table 1 below with references and/or the NCBI reference sequence. Those lacking references were determined by our laboratory.

### 2.5 Priority PAH detection using gas chromatography/mass spectrometry analysis

A WSP aliquot with 20 μl of a 10 mg/mL stock was extracted with 1.0 mL of isooctane and sonicated for 15 min. Following sonication, 0.5 mL was removed and added to a vial glass vial and dried completely under a stream of N_2_. Fifty μl of the sample in isooctane solvent was then added to the column and run under standard GC/MS conditions with an established protocol in the Mass Spectrometry Core facility in the Colorado Skaggs School of Pharmacy (Tooker et al., 2021). Analysis was conducted on an Agilent GC/MS using a published and validated method for PAHs which includes 18 total analytes (16 of which are the EPA priority PAHs). We did not observe any chemicals (e.g., PAHs, metals) in the control air filtered sample.The GC/MS data was also entered into the National Institute of Standards and Technology (NIST) database to identify compounds of interest in the WSP that had a match factor >80, which indicates similarity to the structures in the database found in Supplementary Table 2, Tab 1.

### 2.6 ICP-MS analysis

One microliter aliquots of 10 mg/mL WSP solution were diluted 1:200 in 75% HCl and 25% HNO_3_ (aqua regia) and left to incubate overnight at room temperature for wet digestion. Samples were then diluted 1:50 in 1.5% HNO3 for instrumental analyses. Inductively coupled plasma-mass spectrometry analyses were done on a NexION 2000B single quadrupole ICP-MS (Perkin-Elmer, Waltham, MA) equipped with a Meinhard nebulizer and a cyclonic spray chamber. Prior to use, the instrument was tuned with a solution of one part per billion (ppb) Li, Ce, In, Pb and U to optimize sensitivity and robustness. Samples were analyzed via total quantitative analysis scanning from m/z ratios of 7 to 238. To quantify the concentrations in the sample, the instrument was calibrated to a one-point external standard with a mixture containing 10-ppb each of 40 elements. Data are presented in Supplementary Table 2, Tab 2 as total ng/mL for each metal.

### 2.7 IL-6 ELISA

The human IL-6 Duoset ELISA kit (Bio-Techne/R&D systems, Minneapolis, MN) was used following the manufacturer’s instructions. Media was collected and immediately frozen at −80 °C from studies with the BEAS-2B cells.

### 2.8 Scalpel-loaded dye-transfer assay (SL/DT)

Following treatment as described above, BEAS-2B cells were washed three times with phosphate buffered saline (PBS), followed by adding Lucifer Yellow Dye (Sigma-Aldrich, St. Louis) at 1 mg/mL in PBS to the cells prior to using a scalpel to load the cells in three areas of each culture plate. The dye was allowed to transfer through gap junctions for three minutes followed by three more PBS washes and fixing the cells with 4% formalin (Sigma-Aldrich). Cells were imaged using an Eclipse Ti-S microscope at 100X equipped with a DS-QiMc camera (Nikon Instruments, Melville, NY). The area of dye spread in the image was quantified using ImageJ software (http://imagej.nih.gov/ij/) and analyzed by comparing treated cells to control for the final fraction of control (FOC) percentages. Three scalpel incisions/images were made per dish, and there were three dishes per treatment, for a total of n=9 per experiment; each experiment was repeated three times. This methodology can be found in (Upham, 2011).

### 2.9 Statistical analysis

Graphpad Prism Software (10.3.1) was used for all statistical analyses and graphing. A one-way ANOVA was used to compare variances among the averages of the different treatments followed by Tukey’s multiple comparisons for all comparisons except gene expression for *CYP1A1*, *CYP1B1, GJA1*, *GJB2*, and *EREG*. For these comparisons, a Welch’s t-test (two-tailed) was used. P < 0.05 was considered significant for all comparisons and tests. All studies were repeated three times with an n=2-3 per study.

## 3 Results

### 3.1 Cytotoxicity in response to WSPs in the BEAS-2B cells

**FIGURE 1:**
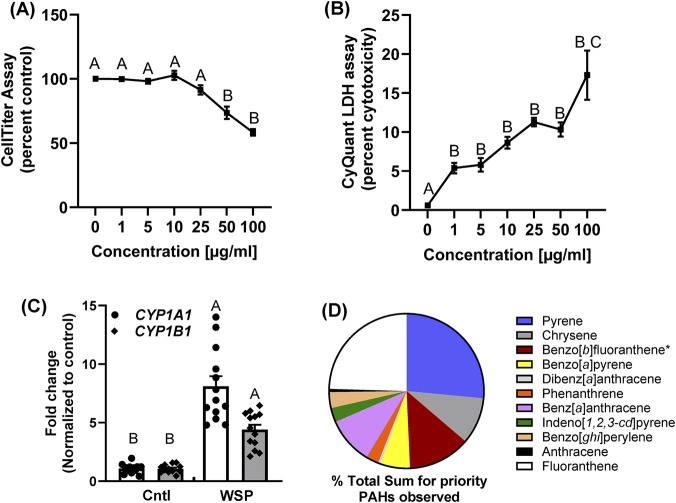
Cytotoxicity and cytochrome p450 gene expression following wood smoke particle (WSP) exposure to human BEAS-2B cells as well as the total sum of PAHs measured in the WSP. In **(A)** BEAS-2B cells were tested for cytotoxicity using Celltiter assay for 24 h exposures to varying concentrations of WSP (0–100 μg/mL) with an n=3-9 per treatment group and repeated three times; mean ± SEM presented. B significantly differs from A (p < 0.05) including vehicle control (0 μg/mL, dH_2_O) and all other doses. **(B)** BEAS-2B cells were tested for cytotoxicity using the CyQuant LDH assay with the same concentrations noted for **(A)**. B significantly differs from A (p < 0.05), vehicle control; C (100 μg/mL) significantly differs (p < 0.05) from all other doses. **(C)** BEAS-2B cells were treated with 5 μg/mL (1.136 μg/cm^2^) WSP or vehicle control for 24 h, RNA isolated followed by reverse transcription for cDNA (Bauer et al., 2024). mRNA analysis was done using standard RT-PCR with Sybr green and amplified with primers specific to *CYP1A1* and *CYP1B1*. Samples were normalized to the expression of 18S rRNA using the comparative CT method (Bauer et al., 2024). Each treatment group had n=3 and was repeated three times; mean ± SEM presented as well as individual samples. A significantly differs from B (p < 0.05) vehicle control. **(D)** Pie chart for the sum of total priority PAHs observed in the WSP (see Table 1 for concentrations) analyzed by GC/MS. *Benzo[*b*]fluoranthene and benzo[*k*]fluoranthene could not be resolved by GC/MS.

Twenty-four hours following exposure to the WSP, we evaluated cytotoxicity (Figures 1A,B). We observed minimal cytotoxicity at ≤ 25 μg/mL WSP that significantly increased at ≥50 μg/mL, measured by two cytotoxicity assays. Based on the cytotoxicity observed with the doses above 25 μg/mL, we choose to use 5 and 25 μg/mL (1.136 and 5.68 μg/cm^2^, respectively) as the dose range for our subsequent studies as well as based on previous studies, as noted in the methods section above.

### 3.2 PAH characterization and ICP-MS analysis of metals on WSP

To understand the potential role of PAHs in these WSP, we assessed the BEAS-2B cell mRNA expression of two cytochrome p450 enzymes (*CYP1A1* and *CYP1B1*) that are known to metabolize PAHs, such as benzo[*a*]pyrene (B[*a* ]P) (Walsh et al., 2013; Geier et al., 2018). In Figure 1C, WSP’s induce both the *CYP1A1* and *CYP1B1* genes after 24 h exposure to 5 μg/mL WSP. Further, we evaluated the U.S. EPA priority PAHs on these WSP using a GC/MS method that we previously established (Tooker et al., 2021), Figure 1D and Table 1. Many of the PAHs identified were the same PAHs that we and others evaluated individually or in combustion mixtures such as burn pit or wildfire PM (Bauer et al., 2024; Kim et al., 2021; Kim et al., 2018; Navarro et al., 2019a; Osgood et al., 2017), including B[*a*]P, fluoranthene, phenanthrene, anthracene, and pyrene. In addition, the measurements were also comparable to other studies (Kim et al., 2018; Kim et al., 2021). In Supplementary Table 2, Tab 1, we also evaluated unknown compounds from the National Institute of Standards and Technology (NIST) database, with match factors >80 that are PAH derivatives and additional compound classes observed in other similar WSP and combustion studies (Corsini et al., 2013; Jen et al., 2019; Kim et al., 2021). These additional classes of compounds identified from the NIST database include aliphatics, aldehydes, among others, however it is not clear if these identified compounds in this and other studies have adverse health effects. Supplementary Table 2, Tab 2 shows measured metals in WSP using ICP-MS with established and standardized methods. We did not observe any notable metals in this analysis.

**TABLE 1:** Gas chromatography/Mass spectrometry (GC/MS) analysis of PAHs in the wood smoke particles generated from Douglas fir wood. A standard methodology was utilized and is described in the Methods and Materials as well as Tooker et al. (2021). Percent total sum of priority PAHs in column 4 is depicted in Figure 1D.

Name of PAH	Final concentration (ng/mL)	Final concentration (ng/mg WSP particles)	% Total sum priority PAHs
Acenaphthylene	ND	ND	ND
Naphthalene	<LOQ	<LOQ	<LOQ
Pyrene	2130.78	213.08	26.50
Chrysene	785.68	78.57	9.77
Benzo[*b*]fluoranthene and Benzo[*k*]fluoranthene^a^	1046.73	104.67	13.02
Benzo[*a*]pyrene	502.82	50.28	6.25
Dibenz[*a,h*]anthracene	39.74	3.97	0.49
Phenanthrene	209.51	20.95	2.61
Benz[a]anthracene	793.22	79.32	9.87
Indeno[*1,2,3-cd*]pyrene	238.27	23.83	2.96
Benzo[*ghi*]perylene	267.50	26.75	3.33
Acenaphthene	ND	ND	ND
Fluorene	ND	ND	ND
Anthracene	54.03	5.40	0.67
Fluoranthene	1972.34	197.23	24.53
2-Methylnaphthalene	ND	ND	ND
1-Methylnaphthalene	ND	ND	ND

^a^ Benzo[*b*]fluoranthene and Benzo[*k*]fluoranthene could not be resolved.

### 3.3 Cytokine and bioactive lipid pathways involved in response to WSP

**FIGURE 2:**
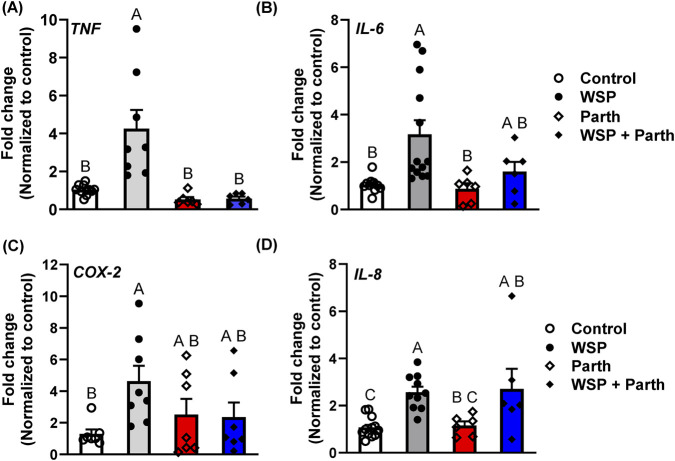
Wood smoke particle-exposed BEAS-2B cells increase mRNA expression of pro- inflammatory cytokines and bioactive lipid pathways. In these studies, we exposed the BEAS-2B cells to 5 μg/mL WSP or vehicle control for 24 h. Cells were pretreated with parthenolide (Tocris; 5 μM) for 1 h prior to WSP exposure or control. RNA was isolated and cDNA amplified as noted in Figure 1 with primers specific to **(A)** *TNF*, **(B)** *IL-6,* **(C)** *COX-2*, and **(D)** *IL-8* (see Supplementary Table 2 for primer sequences). Each treatment group had n=2-3 and was repeated three times; mean ± SEM presented as well as individual samples. For *TNF*, A significantly differs from B (p < 0.05). For *IL-6* and *COX-2*, A significantly differs from B (p < 0.05); AB does not differ from either vehicle control nor WSP treatment. For *IL-8*, A significantly differs from C (p < 0.05); BC significantly differs from WSP treatment but not vehicle control (p < 0.05); AB does not differ from WSP treatment nor parthenolide.

Based on previous studies, we examined the effects of WSP on mRNA expression of *TNF*, *IL-6*, *IL-8*, and *COX-2* 24 h after exposure to 5 μg/mL WSP in the BEAS-2B cells. All four of these cytokines or lipid mediator pathways were significantly elevated above the control in response to WSP. In the presence of a pan-inflammatory inhibitor (parthenolide), *TNF* mRNA expression induced by the WSP was suppressed (Figure 2A). However, parthenolide did not significantly suppress the increased expression of *IL6, IL8*, nor *COX-2* in response to WSP (Figures 2B–D). Interestingly, significant IL-6 secretion was observed via ELISA analysis in response to 5 μg/mL WSP following 24 h exposure; the 5 μg/mL WSP exposure was significantly suppressed by parthenolide (Supplementary Figure 3).

### 3.4 BEAS-2B-expressed connexin mRNA expression and GJIC activity in response to WSP

**FIGURE 3:**
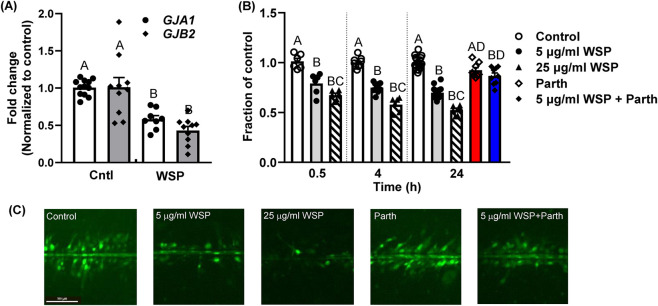
Critical gap junction genes and gap junction activity are inhibited in response to wood smoke particles: parthenolide reverses that inhibition. BEAS-2B cells were exposed to 5 μg/mL WSP or vehicle control for 24 h. **(A)** *GJA1* and *GJB2* mRNA expression are significantly reduced in response to 24 h exposure of 5 μg/mL WSP. RNA was isolated, and cDNA amplified with primers specific to *GJA1* and *GJB2* (see Supplementary Table 2 for primer sequences). Each treatment group had n=3 and was repeated twice. B significantly differs from A (p < 0.05). **(B)** The SL/DT assay was used for these studies to evaluate GJIC activity and was significantly reduced in response to both the 5 and 25 μg/mL WSP exposure at 30 min, 4 h, and 24 h time points, indicating that this response was rapid and prolonged. Parthenolide significantly prevented 5 μg/mL WSP-suppressed GJIC at 24 h. Parthenolide (Tocris; 5 μM) was pretreated for 1 h prior to WSP exposure for these studies. Treatment groups were compared to the vehicle control for final fraction of control (FOC) percentages. A total of n=9 per experiment repeated three times; mean ± SEM presented as well as individual samples. B significantly differs from A (p < 0.05); C (25 μg/mL dose) significantly differs from 5 μg/mL dose; AD, parthenolide significantly differs from the 5 μg/mL dose, but does not differ from vehicle control; BD, WSP + parthenolide differs from vehicle control and 5 μg/mL WSP. **(C)** Representative images for 24 h time point for each treatment. Bar indicates 100 μm.

We tested both the mRNA expression of connexin genes and the inhibition of gap junction activity, which is a common characteristic in premalignant lung cells during tumor promotion. In these novel studies, Figure 3A shows a reduction in expression of two gap junction genes (*GJA1* and *GJB2*) in response to 5 μg/mL WSP in the BEAS-2B cells. We also determined the GJIC activity using our well established SL/DT transfer assay (Upham, 2011). WSP significantly reduced GJIC at 5 and 25 μg/mL in the BEAS-2B cells at 30 min, 4 h, and 24 h time points (Figures 3B,C). We also observed that parthenolide prevented inhibition of GJIC in BEAS-2B cells treated with WSP (5 μg/mL) after 24 h (Figure 3B). This observation suggests that inhibition of GJIC was potentially associated in some way with the inflammatory mediator response. A similar response to parthenolide was observed in a mouse alveolar type II cell line (C10 cells) with a binary PAH-mixture (Romo et al., 2019).

### 3.5 An additional premalignant (tumor-promoting) endpoint: a growth factor (epiregulin)

Epiregulin is an EGFR and ERBB4 ligand that has been recognized as a potential target for interventions for lung cancer (Sunaga et al., 2024) and is also under the sustaining proliferative signaling hallmark of cancer (Hanahan and Weinberg, 2011). WSP (5 μg/mL) increased epiregulin mRNA expression in the BEAS-2B cells, similar to the response observed in mouse lung following *in vivo* exposure to a PAH mixture (Bauer et al., 2024) (Supplementary Figure 4).

## 4 Discussion

Several recent studies present evidence that air pollution, specifically PM (Diaz-Gay et al., 2025; Hill et al., 2023), is associated with an increased risk of the development of LUAD, the most prevalent type of lung cancer (Barta et al., 2019; Diaz-Gay et al., 2025; Hill et al., 2023). The causes of LUAD include cigarette smoking, but it is also the primary type of lung cancer associated with environmental and occupational etiologies such as air pollution (IARC, 2016; Barta et al., 2019; Bade and Dela Cruz, 2020). Studies to understand how these particulates can cause lung cancer are lacking. Thus, these unknown mechanisms need to be carefully evaluated at the early stages during premalignancy (e.g. tumor promoting events) to fully understand how the exposures to these particulates have the potential to lead to LUAD that typically develops over many years until diagnosis (Bade and Dela Cruz, 2020). These studies herein provide evidence that WSP, a surrogate for wildfire smoke PM, increased several pro- inflammatory pathways and epiregulin, as well as inhibited gap junctions in the BEAS-2B cells. Additionally, in the presence of parthenolide, several of these responses were suppressed.

We previously determined that specific PAH species (e.g., fluoranthene) induced numerous inflammation markers (increased pro-tumorigenic cytokines (e.g., tumor necrosis factor (TNF), interleukin (IL)6, KC (hIL-8)), pro-inflammatory lipid mediator pathways (e.g., cyclooxygenase (COX)2 pathway), epiregulin, and dysregulation of GJIC in our lung models (Bauer et al., 2022; Romo et al., 2019; Siegrist et al., 2019). When evaluated *in vivo* (Bauer et al., 2024), these same PAHs were tumor promoters, and the same endpoints (*Cx43*, *Ereg*) responded similarly to the *in vitro* models. We also observed a similar response in this WSP model that supports a potential link to premalignant processes in the lung. Increases in some of these cytokines (IL-6, IL-8) have been observed in other WSP models (Bolling et al., 2012; Corsini et al., 2013), which complements our results, but importantly, these other studies used different sources of wood, higher doses to elicit these responses, and were not associating these responses with any specific lung disease.

We are the first group to report that WSP suppress gap junction activity, increase EREG mRNA expression, and demonstrate parthenolide’s interventional potential. Our results showing that WSP-exposed cells inhibit GJIC are also supported by numerous other studies that demonstrate the importance of altered cell-to-cell communication through gap junctions in early-stage (premalignant, tumor promotion) cancer (Tai et al., 2007; Upham, 2011; Upham et al., 2008). The results found here with parthenolide as an intervention, are intriguing given its known anti-cancer and anti-inflammatory properties. Parthenolide is a natural product derived from the medicinal plant feverfew (*Tanacetum parthenium*) used since the 1500’s for migraines and other diseases associated with inflammation (e.g., arthritis, etc). The major mechanisms of action for parthenolide are as an inhibitor of the NFκB, AP-1, and MAPK pathways (Sztiller-Sikorska and Czyz, 2020; Ghantous et al., 2013; Mathema et al., 2012). Interestingly, it also inhibits STAT3 which is downstream of IL-6 signaling (Liu et al., 2018; Sztiller-Sikorska and Czyz, 2020). In addition, parthenolide degrades MDM2 promoting p53 activity and reduces glutathione, increasing oxidative stress (Sztiller-Sikorska and Czyz, 2020). Our results support the need for more studies to investigate the intervention potential of parthenolide in response to WSP, such as with the other pathways we evaluated.

EREG, a growth factor receptor ligand known to be increased during lung tumor promotion in mice and in human LUAD, is involved in wound healing, proliferation, and other events linked to tumor development in the lung, but is expressed at low levels in healthy lung tissue (Sunaga et al., 2024; Riese and Cullum, 2014). We previously demonstrated that EREG induced wound healing in BEAS-2B cells (Bauer et al., 2017). Ereg has also been shown to influence lung remodeling during lung fibrosis, a chronic lung disease with altered homeostasis, thus EREG has links to multiple lung diseases, most prominently in lung cancer (Ezaddoustdar et al., 2025; Sunaga et al., 2024). Additionally, epiregulin is upregulated in response to some of the cytokines (e.g., IL-6, TNF) and pro-inflammatory pathways (COX-2 pathway) involved in tumor promoting events (Sunaga et al., 2013; Bazzani et al., 2017; Bauer et al., 2017; Murakami et al., 2013; Ezaddoustdar et al., 2025). Because EREG is an EGFR ligand and EGFR mutations are known drivers for non-smoking related LUAD (Hill et al., 2023; Diaz-Gay et al., 2025), we plan to evaluate EGFR signaling in the future, as well as ERB4, the other EREG receptor (Sunaga et al., 2024). In addition, connexins (e.g., CX43) can be inhibited by MAPK activation, such as p38 or ERK1/2 (Sunaga et al., 2024; Osgood et al., 2017), which is downstream of EREG/EGFR activation (Liu et al., 2024).

We recognize some limitations of this study. 1) Except for the assessment of GJIC, a limited number of time points were used to assess our selected endpoints. For example, given the reduction in IL-6 secretion at 24 h in response to parthenolide, but only a trend in suppression of *IL-6* mRNA expression due to parthenolide at 24 h, we would predict an earlier point would demonstrate mRNA differences. 2) We did not definitively link PAHs with the increase of *CYP1A1* and *CYP1B1* genes as other toxicants on the particles can potentially elicit increases in these enzymes (Walsh et al., 2013). We did show that PAHs are present on the WSP at varying levels but did not fully characterize all the other possible toxicants present on those particles and understand these other toxicants are possibly influencing the responses observed. As such, we cannot say that there is a direct correlation with the observed cellular effects, however for some of these individual PAHs, such as fluoranthene and pyrene, known adverse cellular events occur in epithelial cells of the lung and liver, such as inhibition of GJIC, activation of mitogen activated protein kinases (p38 and ERK1/2 MAPK), and increases in inflammatory mediators, as noted above (Bauer et al., 2022; Bauer et al., 2018; Osgood et al., 2017; Osgood et al., 2013; Siegrist et al., 2019; Upham et al., 2008; Upham et al., 1994; Upham et al., 1998; Weis et al., 1998). In addition, recent evidence from Zhang et al., 2024 demonstrates that household air pollution in a specific region of China has carcinogenic PAH profiles that are associated with higher lung cancer incidence in those same regions (Zhang et al., 2024). Further, studies in Guangzhou city, China showed that four higher molecular weight PAHs (e.g., benzo[*b*]fluoranthene) were associated with respiratory diseases and when used in in vitro studies with the BEAS-2B cells, were linked to inflammation, DNA damage, and apoptosis (Jiang et al., 2023). Lastly, in the future, AhR inhibition will also be tested to evaluate the role of Ahr in response to these WSP exposures given the link between many PAHs and downstream Ahr signaling events, similar to published literature (Vondracek et al., 2007). 3) Air liquid interface cultures should be used in the future as a more physiological model to test these exposures, since only a single human lung cell line was used. Thus, extrapolation to human health risks should be made cautiously without additional studies. Importantly, the BEAS-2B model is a well-established model for lung toxicant studies used by numerous groups, including from the National Institute for Occupational Safety and Health (NIOSH)(Sargent et al., 2012; Siegrist et al., 2014; Stueckle et al., 2012). 4) Acute exposures were tested in these studies and in the future, more frequent and longer exposures should be tested. In addition, these endpoints should be evaluated in animal models and if possible, in wildland firefighter populations. 5) These are only a few endpoints and a more global evaluation using multiple omics methods should be used to fully determine a profile of the premalignant effects that occur in response to WSP exposure.

Collectively, these results provide evidence for a new mechanistic understanding of how WSP contribute to lung diseases, such as lung cancer, through a link between inflammation and gap junctions that are both potentially associated with EREG and hence the EGFR and/or ERB4 signaling (Sunaga et al., 2024). These findings are similar to our published results on PAH exposed lung cells. Considering that PAHs we previously studied were also measured in the Douglas fir WSP, our findings suggest that the PAHs were likely key components involved in the observed results, but more studies are needed to validate and extrapolate to determine human health risks. Further, these results indicate the potential to prevent these adverse health outcomes in populations exposed to wildfire PM with natural products such as parthenolide. Future studies are critical to evaluate these pathways in both *in vivo* models and human populations to better understand the exposures, susceptibilities, and possibilities for future interventions.

## Data Availability

The original contributions presented in the study are included in the article/Supplementary Material, further inquiries can be directed to the corresponding author.
