# Supplementary material for: Wood smoke particles elicit events associated with adverse effects in human lung epithelial cells
Source: Front Toxicol. 2026 Mar 20;8:1671925. doi: 10.3389/ftox.2026.1671925 (PMC13047562; doi:10.3389/ftox.2026.1671925)
Supplement: Supplementary file 1 [file DataSheet1.docx]

Supplementary Material

# Supplementary Figures and Tables

## Supplementary Figures

Supplementary Figure 1. Schematic of the experimental setup at Colorado State University to characterize emissions from wood combustion. Colored words represent sampling instruments used for this work. *Reprinted with permission from Van Zyl et al.,* *Environ. Sci. Technol. 2019, 53, 8, 4648–4656; doi:10.1021/acs.est.9b00235. Copyright 2019 American Chemical Society.*


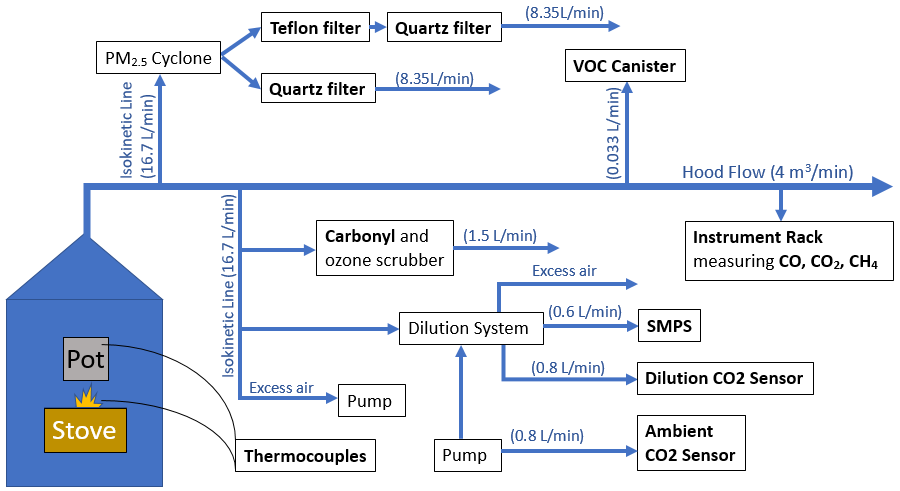


**Teflon Filter**

**Instrument Rack for CO, CO_2_**

**SMPS**

**Dilution CO_2_ sensor**

**Ambient CO_2_ sensor**

**Thermocouples**

**
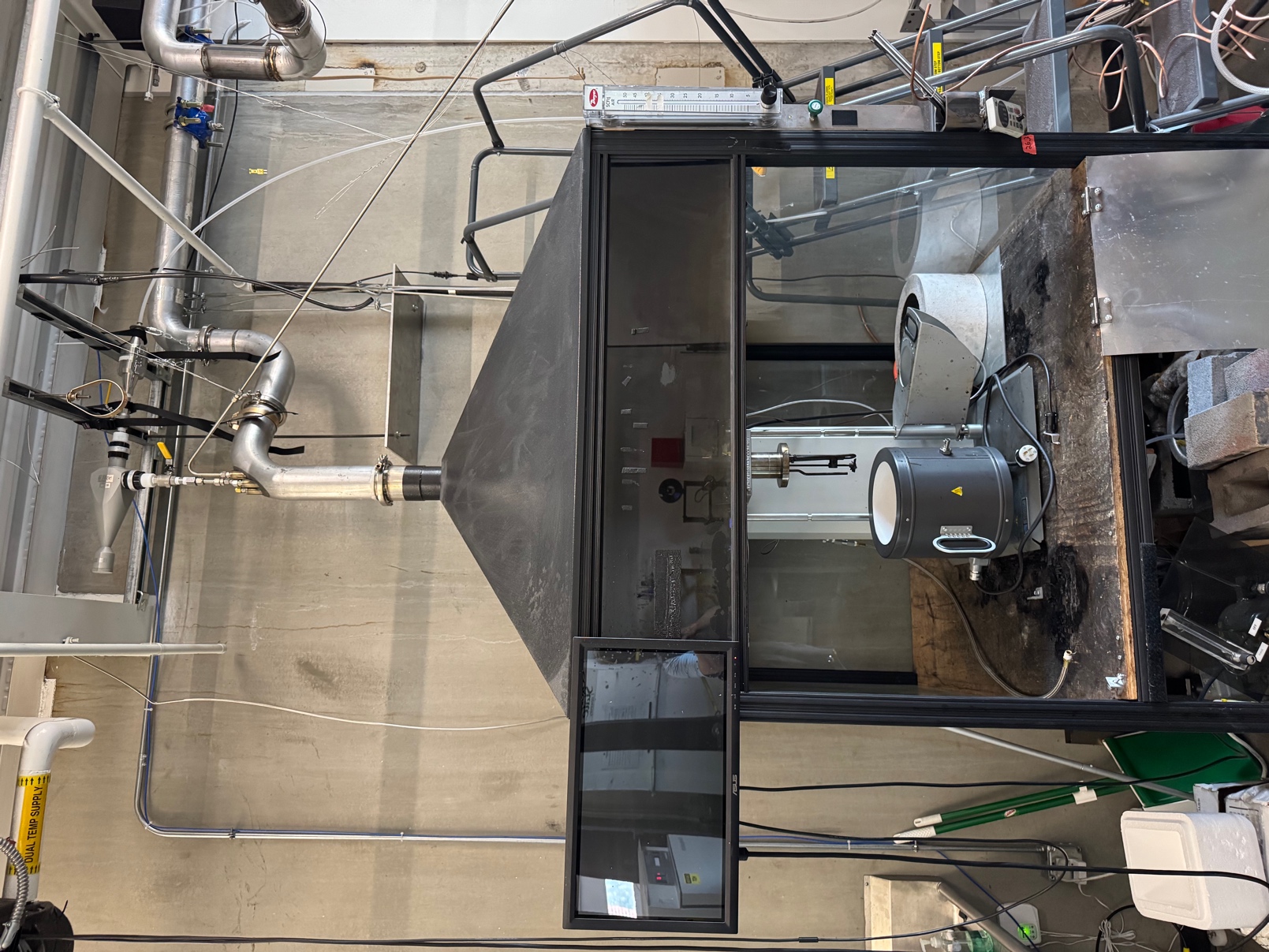
Supplementary Figure 2.** Photo of the emissions testing hood at Colorado State University. Hood flow is controlled by the touch-screen instrument panel at the left. Isokinetic sampling lines for PM sampling are shown at top.

**
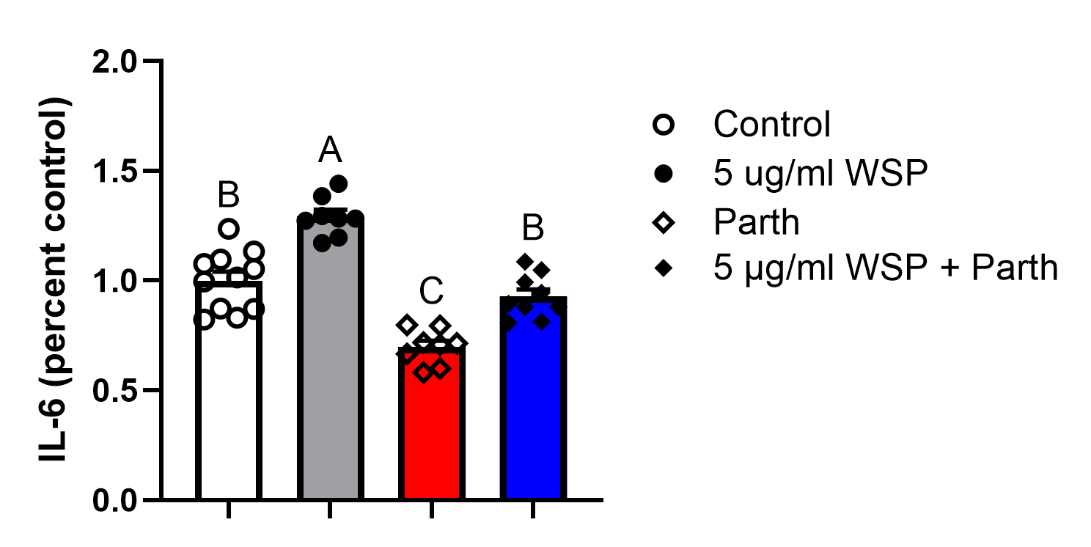
**

**Supplementary Figure 3.** IL-6 secretion presented as percent control for BEAS-2B cells exposed to 5 WSP versus control. Parthenolide significantly prevented 5 μg/ml WSP-increased IL-6 secretion at 24 h. Parthenolide (Tocris; 5 μM) was pretreated for 1 h prior to WSP exposure for these studies. Data presented as mean ± SEM as well as individual data points; n=3, repeated three times. A significantly differs (p<0.05) from B vehicle control; C significantly differs (p<0.05) from A and B.

**
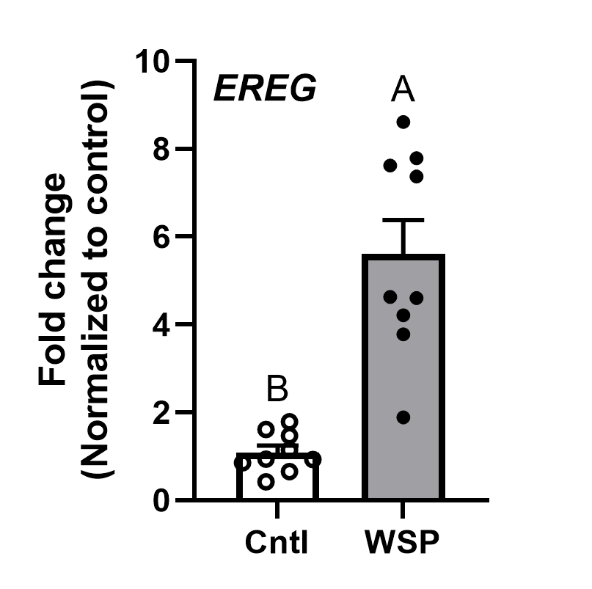
**

**Supplementary Figure 4.** **Epiregulin mRNA expression following 24 h exposure to 5 μg/ml WSP in BEAS-2B cells.** mRNA expression was evaluated with primers specific to *EREG*. Each treatment group had n=3 and was repeated three times. A significantly differs (p<0.05) from B vehicle control.

## 2.2 Supplementary Tables

**Supplementary Table 1: Primer sequences used for qRT-PCR.**

| **Human gene name** | **Forward primer 5′-3′** | **Reverse Primer 5′-3′** | **Ref. or NCBI reference sequence** |
| --- | --- | --- | --- |
| *18S* | ATTCGTATTGCGCCGCTAGAG | GGCATCGTTTATGGTCGGAAC | XR_007090847.1 |
| *CYP1A1* | TCGGCCACGGAGTTTCTTC | GGTCAGCATGTGCCCAATCA | Pinkston et al, 2020 |
| *CYP1B1* | AAGTTCTTGAGGCACTGCGAA | GGCCGGTACGTTCTCCAAAT | Pinkston et al, 2020 |
| *TNF* | GTTGTAGCAAACCCTCAAGCTG | TTGACCTTGGTCTGGTAGGA | NM_000594.4 |
| *IL-8* | AAGAAACCACCGGAAGGAAC | ACTCCTTGGCAAAACTGCAC | Lindbom et al., 2006 |
| *IL-6* | TGGGTCCAGTTGCCTTCTC | GCTTGTTCCTCACTACTCTCA | Lindbom et al., 2006 |
| *COX2* | AACTGCTCAACACCGGAATT | ATTGCATTTCGAAGGAAGG | NM_000963.4 |
| *EREG* | ATCCTGGCATGTGCTAGGGT | GTGCTCCAGAGGTCAGCCAT | Yoshikawa et al, 2013 |
| *GJA1* | TCCCCTCTCGCCTATGTCTC | GTTTTGCTCACTTGCTTGCTTG | Salameh et al, 2014 |
| *GJB2* | CGCAGAGCAAACCGCCCAGA | AGCCTGGCTGCAGGGTGTTG | Dube et al., 2012 |

**Supplementary Table 2: (see Excel file) Additional analysis of the wood smoke particles (WSP) using the NIST database and ICP-MS for metal analysis.** Tab 1. Tentatively identified compounds that were determined using the National Institute of Standards and Technology (NIST) database to identify compounds of interest in the WSP that had a match factor >80, which indicates similarity to the structures in the database. Tab 2. Inductively coupled plasma-mass spectrometry (ICP-MS) data for the metals identified in the WSP in ng/μl.

**References**

Dube E, Dufresne J, Chan PT, Cyr DG. 2012. Epidermal growth factor regulates connexin 43 in the human epididymis: Role of gap junctions in azoospermia. Hum Reprod. 27(8):2285-2296.

Lindbom J, Gustafsson M, Blomqvist G, Dahl A, Gudmundsson A, Swietlicki E, Ljungman AG. 2006. Exposure to wear particles generated from studded tires and pavement induces inflammatory cytokine release from human macrophages. Chem Res Toxicol. 19(4):521-530.

Pinkston R, Zaman H, Hossain E, Penn AL, Noel A. 2020. Cell-specific toxicity of short-term juul aerosol exposure to human bronchial epithelial cells and murine macrophages exposed at the air-liquid interface. Respiratory research. 21(1):269.

Salameh A, Haunschild J, Brauchle P, Peim O, Seidel T, Reitmann M, Kostelka M, Bakhtiary F, Dhein S, Dahnert I. 2014. On the role of the gap junction protein cx43 (gja1) in human cardiac malformations with fallot-pathology. A study on paediatric cardiac specimen. PloS one. 9(4):e95344.

Van Zyl, L., Tryner, J., Bilsback, K. R., Good, N., Hecobian, A., Sullivan, A., et al. (2019). Effects of fuel moisture content on emissions from a rocket-elbow cookstove. *Environ. Sci. Technol.* 53, 4648–4656. doi:10.1021/acs.est.9b00235.

Yoshikawa M, Kojima H, Yaguchi Y, Okada N, Saito H, Moriyama H. 2013. Cholesteatoma fibroblasts promote epithelial cell proliferation through overexpression of epiregulin. PloS one. 8(6):e66725.
